# Supplementary material for: ATPγS substantially defeats the biasing mechanism for kinesin steps
Source: Nat Commun. 2026 Feb 18;17:2891. doi: 10.1038/s41467-026-69573-z (PMC13031914; doi:10.1038/s41467-026-69573-z)
Supplement: Supplementary file 1 — Supplementary Information [file 41467_2026_69573_MOESM1_ESM.pdf]

Supplementary Information

ATPγS substantially defeats the biasing mechanism for kinesin steps

Vishakha Karnawat, Algirdas Toleikis, Nicholas J. Carter, Justin E. Molloy, Robert A. Cross

Centre for Mechanochemical Cell Biology, Warwick Medical School, University of Warwick, Coventry, CV4 7AL, UK

Correspondence: r.a.cross@warwick.ac.uk

Contents

- **Supplementary Figure 1: Cumulative dwell-time distributions and exponential fits at 1 mM ATP**
- **Supplementary Figure 2: Cumulative dwell-time distributions and exponential fits at 1 mM ATPγS**
- **Supplementary Figure 3: Cumulative dwell-time distributions and exponential fits at 1 μM ATP**
- **Supplementary Figure 4: Cumulative dwell-time distributions and exponential fits at 1 μM ATPγS**
- **Supplementary Figure 5: Cumulative dwell-time distributions and exponential fits at 0.9 mM ATP plus 0.1 mM ADP**
- **Supplementary Figure 6: Cumulative dwell-time distributions and exponential fits at 0.9 mM ATPγS plus 0.1 mM ADP**
- **Supplementary Figure 7: Example trapping records under superstall (SS) force at 1 mM ATPγS**
- **Supplementary Figure 8: Distribution of negative amplitudes partitioned into backsteps and detachments**
- **Supplementary Figure 9: Load-dependence of step dwell times in ATP versus ATPγS**

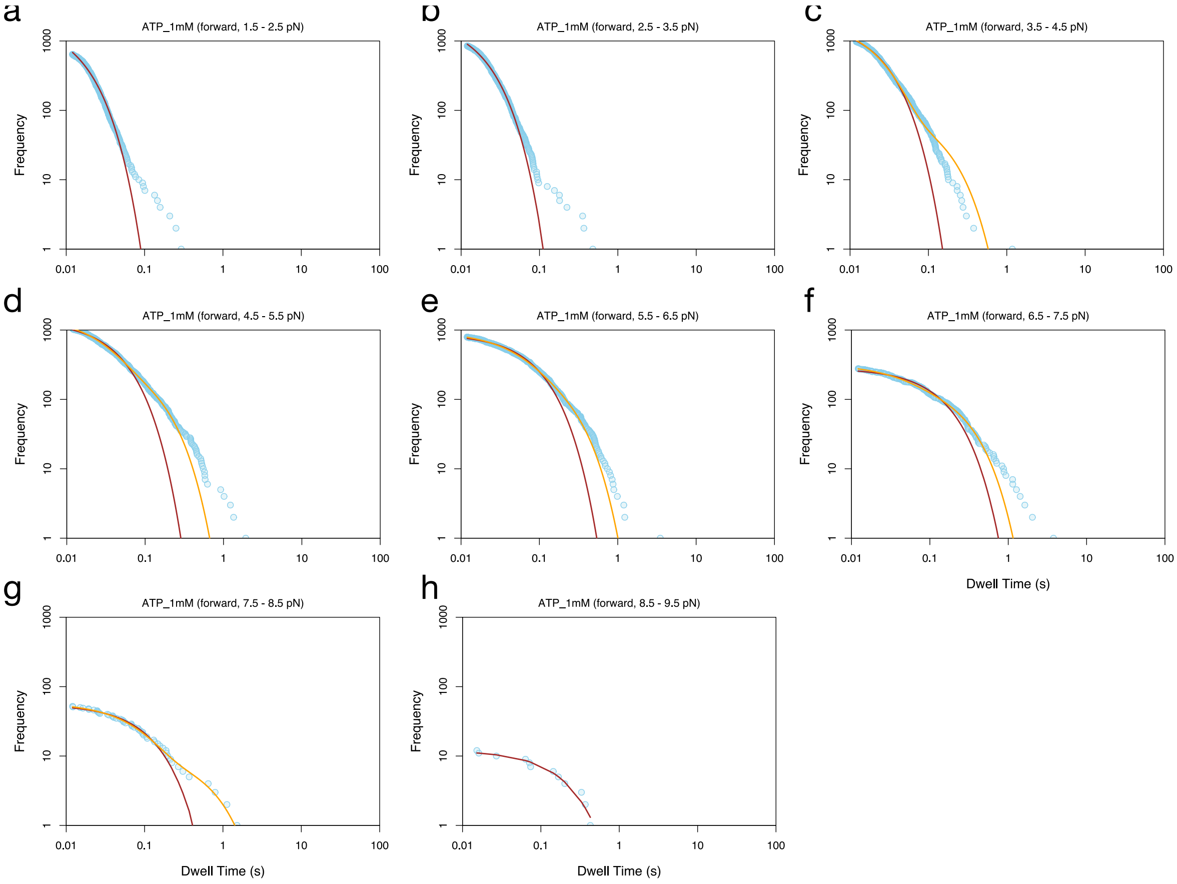
a
ATP_1mM (forward, 1.5 - 2.5 pN)
b
ATP_1mM (forward, 2.5 - 3.5 pN)
c
ATP_1mM (forward, 3.5 - 4.5 pN)
d
ATP_1mM (forward, 4.5 - 5.5 pN)
e
ATP_1mM (forward, 5.5 - 6.5 pN)
f
ATP_1mM (forward, 6.5 - 7.5 pN)
g
ATP_1mM (forward, 7.5 - 8.5 pN)
h
ATP_1mM (forward, 8.5 - 9.5 pN)
Frequency
1
10
100
1000
0.01
0.1
1
10
100
Dwell Time (s)

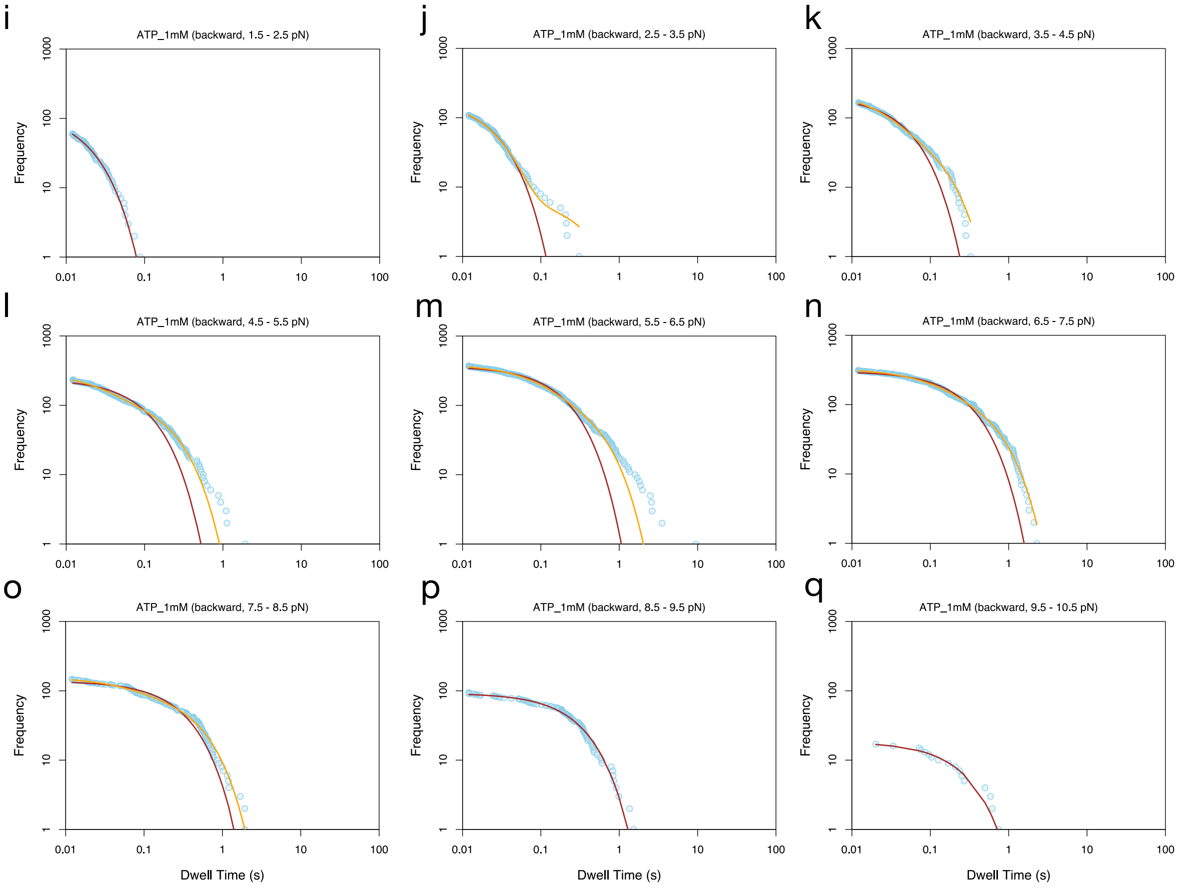
i
ATP_1mM (backward, 1.5 - 2.5 pN)
j
ATP_1mM (backward, 2.5 - 3.5 pN)
k
ATP_1mM (backward, 3.5 - 4.5 pN)
l
ATP_1mM (backward, 4.5 - 5.5 pN)
m
ATP_1mM (backward, 5.5 - 6.5 pN)
n
ATP_1mM (backward, 6.5 - 7.5 pN)
o
ATP_1mM (backward, 7.5 - 8.5 pN)
p
ATP_1mM (backward, 8.5 - 9.5 pN)
q
ATP_1mM (backward, 9.5 - 10.5 pN)
Frequency
1
10
100
1000
0.01
0.1
1
10
100
Dwell Time (s)

Supplementary Figure 1: Cumulative dwell-time distributions and exponential fits at 1 mM ATP.

Cumulative probability plots are shown for all force bins. Forward dwells (top row) and backward dwells (bottom row) were fit with single- and double-exponential models. Blue circles represent experimental data. Solid brown lines indicate single-exponential fits ($y = A\cdot\exp(-b\cdot x)$), and solid orange lines indicate double-exponential fits ($y = A_1\cdot\exp(-b_1\cdot x) + A_2\cdot\exp(-b_2\cdot x)$). Fits were obtained using nonlinear least-squares regression in R. All plots are displayed on log–log axes. Parameters from both single- and double-exponential fits are: **a.** dwell = 1886.82 $e^{(-84.31*load)}$ **b.** dwell = 2025.389 $e^{(-67.862*load)}$ **c.** dwell = 1827.4 $e^{(49.6*load)}$, dwell = 1882.1·$e^{(-58.5*load)}$ + 101.9·$\exp^{(-8.0*load)}$ **d.** dwell = 1354.7 $e^{(-25.3*load)}$, dwell = 1216.5 $e^{(-44.5*load)}$ + 376.4 $e^{(-8.9*load)}$ **e.** dwell = 878.7 $e^{(-12.6*load)}$, dwell = 625.1 $e^{(-22.5*load)}$ + 330.6 $e^{(-5.8*load)}$ **f.** dwell = 280.2 $e^{(-7.5*load)}$, dwell = 148.4 $e^{(-25.3*load)}$ + 175.8$e^{(-4.5*load)}$ g. dwell=55.4 $e^{(-9.5*load)}$, dwell = 48.7 $e^{(-14.3*load)}$ + 10.4 $e^{(-1.7*load)}$ **h.** dwell = 12 $e^{(-5.2*load)}$ i. dwell = 123.3 $e^{(-61.1*load)}$ j. dwell = 186.5 $e^{(-45.0*load)}$, dwell = 189.9 $e^{(-50.9*load)}$ + 7.1 $e^{(-3.2*load)}$ **k.** dwell = 205.3 $e^{(-22.4*load)}$, dwell=168.3 $e^{(-48.7*load)}$ + 81.5 $e^{(-10*load)}$ l. dwell = 237.8 $e^{(-10.4*load)}$, dwell = 165.1 $e^{(-40.7*load)}$ + 139 $e^{(-5.5*load)}$ **m.** dwell = 364.5 $e^{(-5.5*load)}$, dwell = 226.1 $e^{(-12.4*load)}$ + 168.4 $e^{(-2.5*load)}$ **n.** dwell = 300.5 $e^{(-3.6*load)}$, dwell = 155.2 $e^{(-10.2*load)}$ + 173.5 $e^{(-2.0*load)}$ **o.** dwell = 137.2 $e^{(-3.5*load)}$, dwell = 55.1 $e^{(-17.0*load)}$ + 101.2 $e^{(-2.4*load)}$ **p.** dwell = 92.2 $e^{(-3.5*load)}$ **q.** dwell = 18.3 $e^{(-4.0*load)}$

Sample sizes (n) for each force bin: a (1.5–2.5 pN), n = 642; b (2.5–3.5 pN), n = 851; c (3.5–4.5 pN), n = 979; d (4.5–5.5 pN), n = 1035; e (5.5–6.5 pN), n = 794; f (6.5–7.5 pN), n = 278; g (7.5–8.5 pN), n = 52; h (8.5–9.5 pN), n = 12; i (1.5–2.5 pN), n = 60; j (2.5–3.5 pN), n = 109; k (3.5–4.5 pN), n = 166; l (4.5–5.5 pN), n = 234; m (5.5–6.5 pN), n = 370; n (6.5–7.5 pN), n = 312; o (7.5–8.5 pN), n = 147; p (8.5–9.5 pN), n = 94; q (9.5–10.5 pN), n = 17.

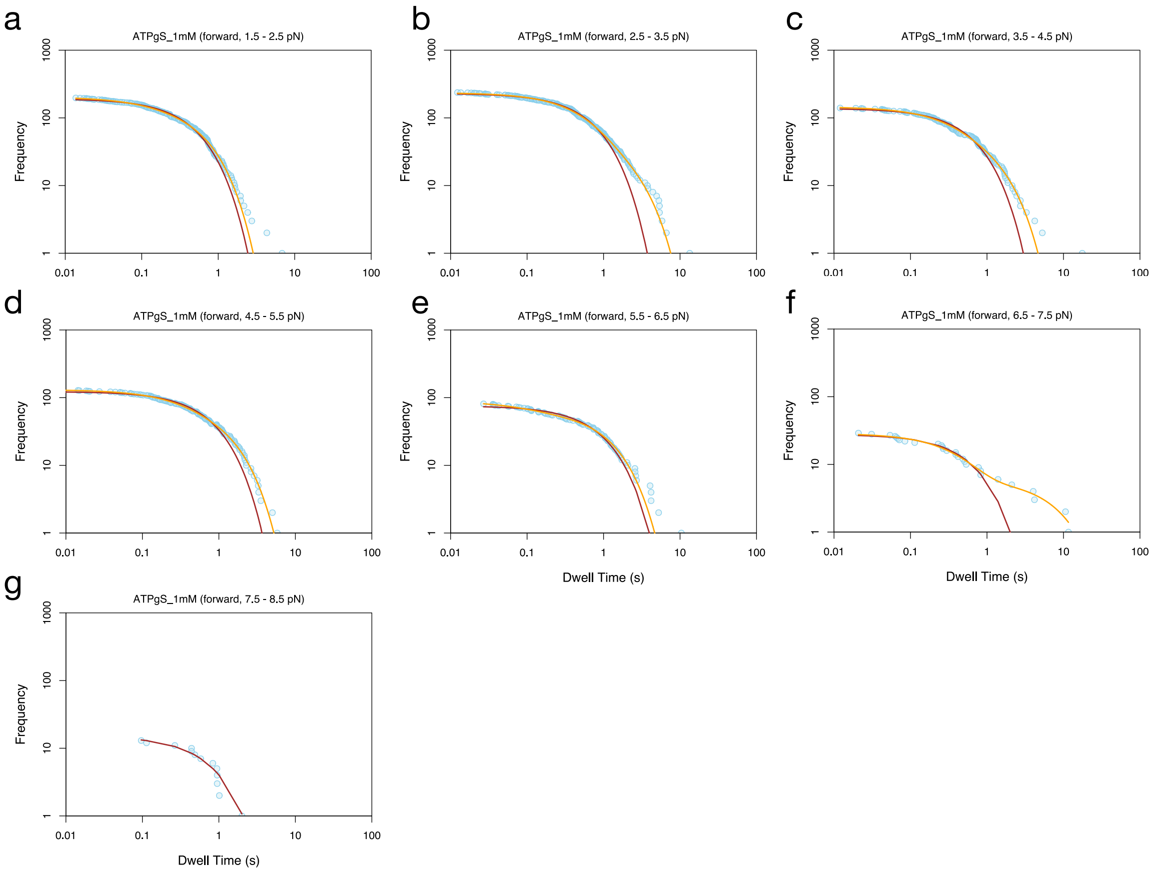
a
ATPgS_1mM (forward, 1.5 - 2.5 pN)
b
ATPgS_1mM (forward, 2.5 - 3.5 pN)
c
ATPgS_1mM (forward, 3.5 - 4.5 pN)
d
ATPgS_1mM (forward, 4.5 - 5.5 pN)
e
ATPgS_1mM (forward, 5.5 - 6.5 pN)
f
ATPgS_1mM (forward, 6.5 - 7.5 pN)
g
ATPgS_1mM (forward, 7.5 - 8.5 pN)
Frequency
1
10
100
1000
0.01
0.1
1
10
100
Dwell Time (s)

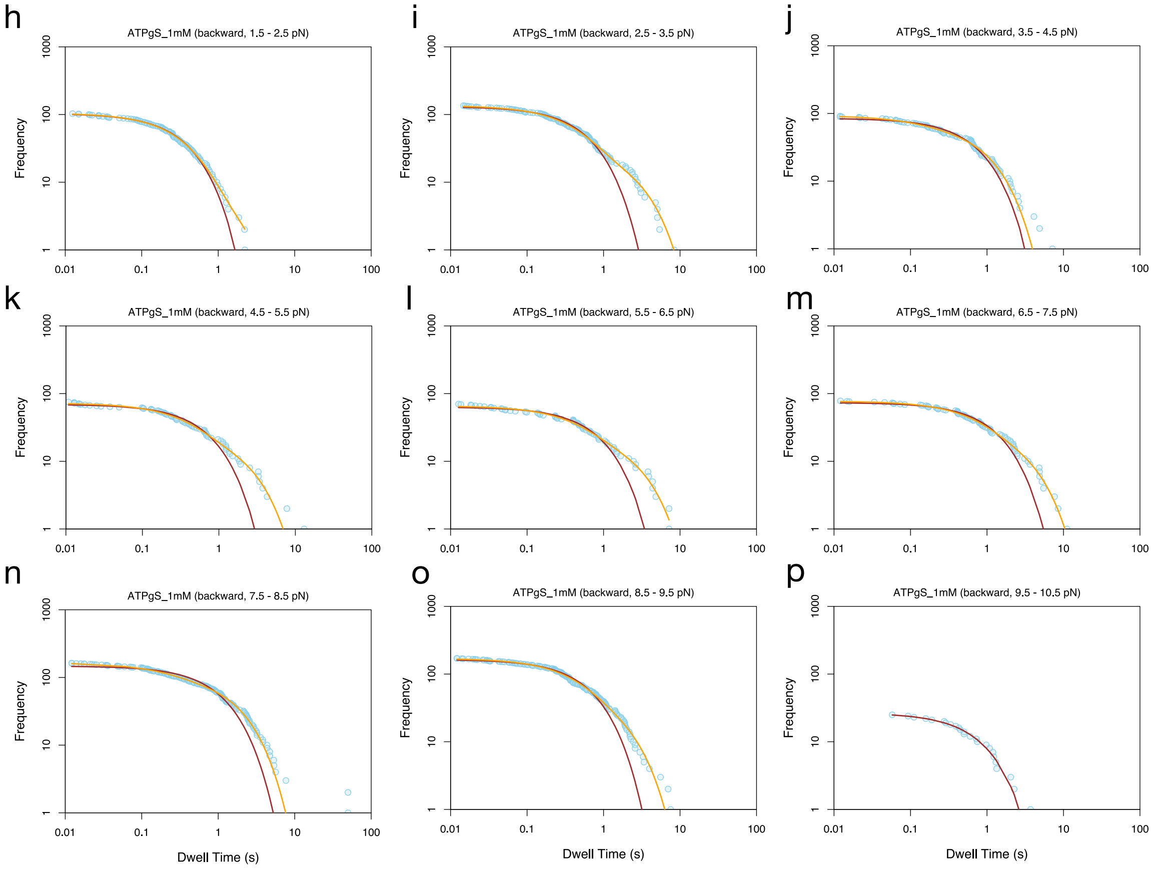
h
ATPgS_1mM (backward, 1.5 - 2.5 pN)
i
ATPgS_1mM (backward, 2.5 - 3.5 pN)
j
ATPgS_1mM (backward, 3.5 - 4.5 pN)
k
ATPgS_1mM (backward, 4.5 - 5.5 pN)
l
ATPgS_1mM (backward, 5.5 - 6.5 pN)
m
ATPgS_1mM (backward, 6.5 - 7.5 pN)
n
ATPgS_1mM (backward, 7.5 - 8.5 pN)
o
ATPgS_1mM (backward, 8.5 - 9.5 pN)
p
ATPgS_1mM (backward, 9.5 - 10.5 pN)
Frequency
1
10
100
1000
0.01
0.1
1
10
100
Dwell Time (s)

Supplementary Figure 2: Cumulative dwell-time distributions and exponential fits at 1 mM ATPγS.

Cumulative probability plots are shown for all force bins. Forward dwells (top row) and backward dwells (bottom row) were fit with single- and double-exponential models. Blue circles represent experimental data. Solid brown lines indicate single-exponential fits (y = A·exp(−b·x)), and solid orange lines indicate double-exponential fits (y = A_1·exp(−b_1·x) + A_2·exp(−b_2·x)). Fits were obtained using nonlinear least-squares regression in R. All plots are displayed on log–log axes. Parameters from both single- and double-exponential fits are: **a.** dwell = 191.1 $e^{(-2.2*load)}$, *dwell = 43.6* $e^{(-9.3*load)}$ + 160.4 $e^{(-1.8*load)}$ **b.** dwell = 229.3 $e^{(-1.5*load)}$, *dwell = 173.1* $e^{(-2.1*load)}$ + 62.7 $e^{(-0.5*load)}$ **c.** dwell = 137.7 $e^{(-1.6*load)}$, *dwell = 73.2* $e^{(-3.6*load)}$ + 72.8 $e^{(-0.9*load)}$ **d.** dwell = 122.8 $e^{(-1.3*load)}$, *dwell = 50.2* $e^{(-3.8*load)}$ + 80.7 $e^{(-0.8*load)}$ ***e.*** *dwell = 75.5* $e^{(-1.1*load)}$, dwell = 23.2 $e^{(-11.6*load)}$ + *65.7* $e^{(-0.9*load)}$ **f.** dwell = 27.7 $e^{(-1.6*load)}$, *dwell = 22.8* $e^{(-2.6*load)}$ + 6.0 $e^{(-0.1*load)}$ ***g.*** *dwell = 15.1* $e^{(-1.3*load)}$ **h.** dwell = 103.6 $e^{(-2.8*load)}$, *dwell = 94.8* $e^{(-3.2*load)}$ + 10.1 $e^{(-0.7*load)}$ ***i.*** *dwell = 130.9* $e^{(-1.7*load)}$, dwell = 104.5 $e^{(-2.6*load)}$ + *31.4* $e^{(-0.4*load)}$ **j.** dwell = 84.7 $e^{(-1.4*load)}$, *dwell = 23.7* $e^{(-8.3*load)}$ + 69.3 $e^{(-1.1*load)}$ ***k.*** *dwell = 69.1* $e^{(-1.4*load)}$, dwell = 46.8 $e^{(-2.8*load)}$ + *25.8* $e^{(-0.5*load)}$ **l.** dwell = 63.2 $e^{(-1.2*load)}$, *dwell = 40.6* $e^{(-2.4*load)}$ + 25.2 $e^{(-0.4*load)}$ **m**. dwell = 73.5 $e^{(-0.8*load)}$, *dwell = 46.1* $e^{(-1.6*load)}$ + 31.2 $e^{(-0.3*load)}$ ***n.*** *dwell = 147.1* $e^{(-1.0*load)}$, dwell = 57.1 $e^{(-5.1*load)}$ + *107.1* $e^{(-0.6*load)}$ **o.** dwell = 163.9 $e^{(-1.6*load)}$, *dwell = 113.2* $e^{(-2.7*load)}$ + 57 $e^{(-0.6*load)}$ ***p.*** *dwell = 26.7* $e^{(-1.2*load)}$. n for each force bin: a (1.5–2.5 pN), n = 197; b (2.5–3.5 pN), n = 237; c (3.5–4.5 pN), n = 140; d (4.5–5.5 pN), n = 129; e (5.5–6.5 pN), n = 81; f (6.5–7.5 pN), n = 29; g (7.5–8.5 pN), n = 13; h (1.5–2.5 pN), n = 103; i (2.5–3.5 pN), n = 135; j (3.5–4.5 pN), n = 91; k (4.5–5.5 pN), n = 75; l (5.5–6.5 pN), n = 70; m (6.5–7.5 pN), n = 78; n (7.5–8.5 pN), n = 163; o (8.5–9.5 pN), n = 171; p (9.5–10.5 pN), n = 25.

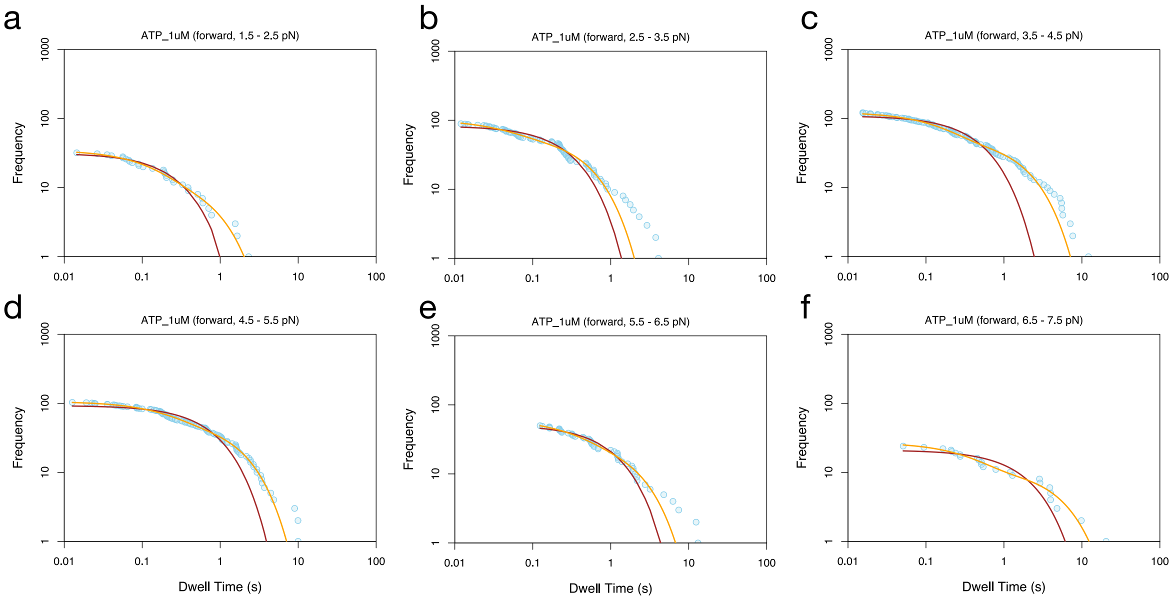
a
ATP_1uM (forward, 1.5 - 2.5 pN)
b
ATP_1uM (forward, 2.5 - 3.5 pN)
c
ATP_1uM (forward, 3.5 - 4.5 pN)
d
ATP_1uM (forward, 4.5 - 5.5 pN)
e
ATP_1uM (forward, 5.5 - 6.5 pN)
f
ATP_1uM (forward, 6.5 - 7.5 pN)
Frequency
1
10
100
1000
0.01
0.1
1
10
100
Dwell Time (s)

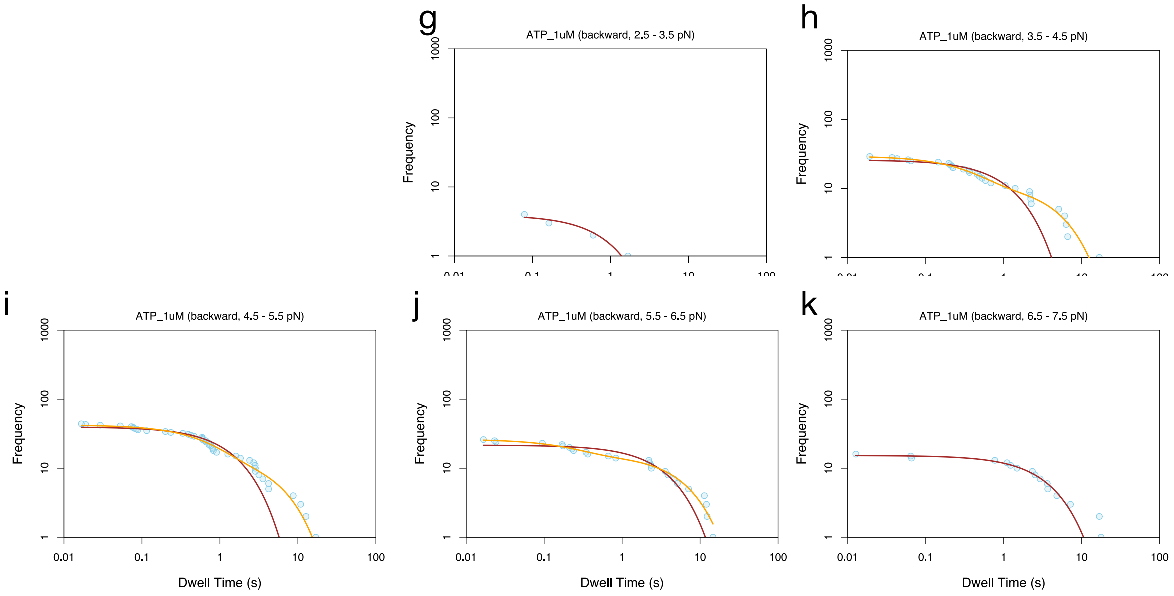
g
ATP_1uM (backward, 2.5 - 3.5 pN)
h
ATP_1uM (backward, 3.5 - 4.5 pN)
i
ATP_1uM (backward, 4.5 - 5.5 pN)
j
ATP_1uM (backward, 5.5 - 6.5 pN)
k
ATP_1uM (backward, 6.5 - 7.5 pN)
Frequency
1
10
100
1000
0.01
0.1
1
10
100
Dwell Time (s)

Supplementary Figure 3: Cumulative dwell-time distributions and exponential fits at 1 μM ATP.

Cumulative probability plots are shown for all force bins. Forward dwells (top row) and backward dwells (bottom row) were fit with single- and double-exponential models. Blue circles represent experimental data. Solid brown lines indicate single-exponential fits (y = A·exp(−b·x)), and solid orange lines indicate double-exponential fits ($y = A_1 \cdot \exp(-b_1 \cdot x) + A_2 \cdot \exp(-b_2 \cdot x)$). Fits were obtained using nonlinear least-squares regression in R. All plots are displayed on log–log axes. Parameters from both single- and double-exponential fits are: **a.** dwell = 31.6 $e^{(-3.3*load)}$, *dwell = 20.8* $e^{(-8.2*load)}$ + 14.3 $e^{(-1.3*load)}$ **b.** *dwell = 82.5* $e^{(-3.2*load)}$, dwell = 41.3 $e^{(-23.4*load)}$ + *60.9* $e^{(-2.0*load)}$ **c.** dwell = 109.7 $e^{(-1.9*load)}$, *dwell = 72.7* $e^{(-6.6*load)}$ + 52.1 $e^{(-0.6*load)}$ **d.** *dwell = 92.5* $e^{(-1.2*load)}$, dwell = 49.8 $e^{(-5.1*load)}$ + *56.4* $e^{(-0.6*load)}$ ***e.*** dwell = 51.2 $e^{(-0.9*load)}$, *dwell = 30.3* $e^{(-3.3*load)}$ + 31.7 $e^{(-0.5*load)}$ **f.** *dwell = 21.0* $e^{(-0.5*load)}$, dwell = 15.8 $e^{(-2.8*load)}$ + *11.3* $e^{(-0.2*load)}$ ***g.*** dwell = 3.9 $e^{(-1.0*load)}$ **h.** *dwell = 25.8* $e^{(-0.8*load)}$, dwell = 17.8 $e^{(-2.8*load)}$ + *11.6* $e^{(-0.2*load)}$ ***i.*** dwell = 39.6 $e^{(-0.6*load)}$, *dwell = 27.0* $e^{(-1.5*load)}$ + 15.4 $e^{(-0.2l*oad)}$ **j.** *dwell = 21.6* $e^{(-0.3*load)}$, dwell = 10.4 $e^{(-4.3*load)}$ + *15.8* $e^{(-0.2*load)}$ ***k.*** dwell = 15.3 $e^{(-0.3*load)}$. n for each force bin: a (1.5–2.5 pN), n = 32; b (2.5–3.5 pN), n = 89; c (3.5–4.5 pN), n = 122; d (4.5–5.5 pN), n = 103; e (5.5–6.5 pN), n = 50; f (6.5–7.5 pN), n = 24; g (2.5–3.5 pN), n = 4; h (3.5–4.5 pN), n = 29; i (4.5–5.5 pN), n = 44; j (5.5–6.5 pN), n = 26; k (6.5–7.5 pN), n = 16.

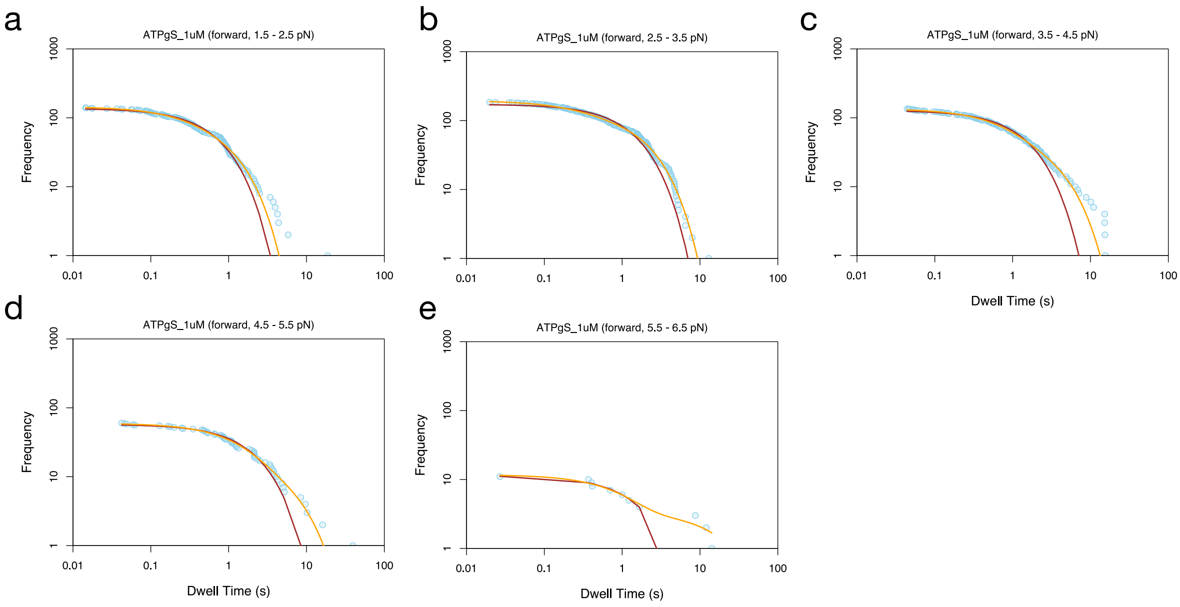
a
ATPgS_1uM (forward, 1.5 - 2.5 pN)
b
ATPgS_1uM (forward, 2.5 - 3.5 pN)
c
ATPgS_1uM (forward, 3.5 - 4.5 pN)
d
ATPgS_1uM (forward, 4.5 - 5.5 pN)
e
ATPgS_1uM (forward, 5.5 - 6.5 pN)
Frequency
Dwell Time (s)

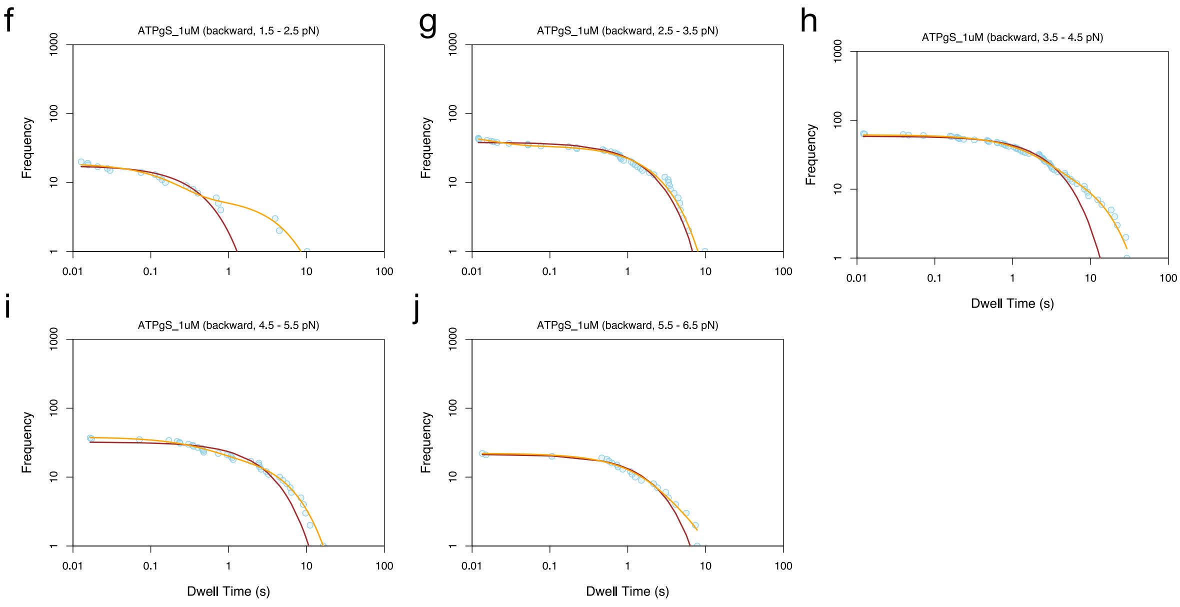
f
ATPgS_1uM (backward, 1.5 - 2.5 pN)
g
ATPgS_1uM (backward, 2.5 - 3.5 pN)
h
ATPgS_1uM (backward, 3.5 - 4.5 pN)
i
ATPgS_1uM (backward, 4.5 - 5.5 pN)
j
ATPgS_1uM (backward, 5.5 - 6.5 pN)
Frequency
Dwell Time (s)

Supplementary Figure 4: Cumulative dwell-time distributions and exponential fits at 1 µM ATPγS.

Cumulative probability plots are shown for all force bins. Forward dwells (top row) and backward dwells (bottom row) were fit with single- and double-exponential models. Blue circles represent experimental data. Solid brown lines indicate single-exponential fits (y = A·exp(−b·x)), and solid orange lines indicate double-exponential fits ($y = A_1 \cdot \exp(-b_1 \cdot x) + A_2 \cdot \exp(-b_2 \cdot x)$). Fits were obtained using nonlinear least-squares regression in R. All plots are displayed on log–log axes. Parameters from both single- and double-exponential fits are: **a.** dwell = 136.7 $e^{(-1.4*load)}$, *dwell = 46.8* $e^{(-4.3*load)}$ + 98.6 $e^{(-1.0*load)}$ **b.** *dwell = 172.4* $e^{(-0.7*load)}$, dwell = 62.4 $e^{(-3.9*load)}$ + *132.2* $e^{(-0.5*load)}$ **c.** dwell = 128 $e^{(-0.7*load)}$, *dwell = 77.3* $e^{(-1.5*load)}$ + 58.2 $e^{(-0.3*load)}$ **d.** *dwell = 57.5* $e^{(-0.5*load)}$, dwell = 40.9 $e^{(-0.8*load)}$ + *18.8* $e^{(-0.2*load)}$ ***e.*** dwell = 11.3 $e^{(-0.6*load)}$, *dwell = 8.4* $e^{(-1.1*load)}$ + 3.4 $e^{(-0.05*load)}$ **f.** dwell = 17.5 $e^{(-2.2*load)}$, *dwell = 12.9* $e^{(-6.2*load)}$ + 6.2 $e^{(-0.2*load)}$ ***g.*** *dwell = 38.3* $e^{(-0.5*load)}$, dwell = 19.5 $e^{(-70.9*load)}$ + *34.8* $e^{(-0.4*load)}$ **h.** dwell = 58.6 $e^{(-0.3*load)}$, *dwell = 39.2* $e^{(-0.6*load)}$ + 22.4 $e^{(-0.1*load)}$ ***i.*** *dwell = 32.2* $e^{(-0.3*load)}$, dwell = 15.8 $e^{(-2.3*load)}$ + *22.4* $e^{(-0.2*load)}$ **j.** dwell = 21.4 $e^{(-0.5*load)}$, *dwell = 15.3* $e^{(-0.8*load)}$ + 6.8 $e^{(-0.2*load)}$ ***k.*** *dwell = 21.4* $e^{(-0.5*load)}$. n for each force bin: a (1.5–2.5 pN), n = 140; b (2.5–3.5 pN), n = 185; c (3.5–4.5 pN), n = 135; d (4.5–5.5 pN), n = 60; e (5.5–6.5 pN), n = 11; f (1.5–2.5 pN), n = 20; g (2.5–3.5 pN), n = 44; h (3.5–4.5 pN), n = 64; i (4.5–5.5 pN), n = 37; j (5.5–6.5 pN), n = 22; k (6.5–7.5 pN), n = 3.

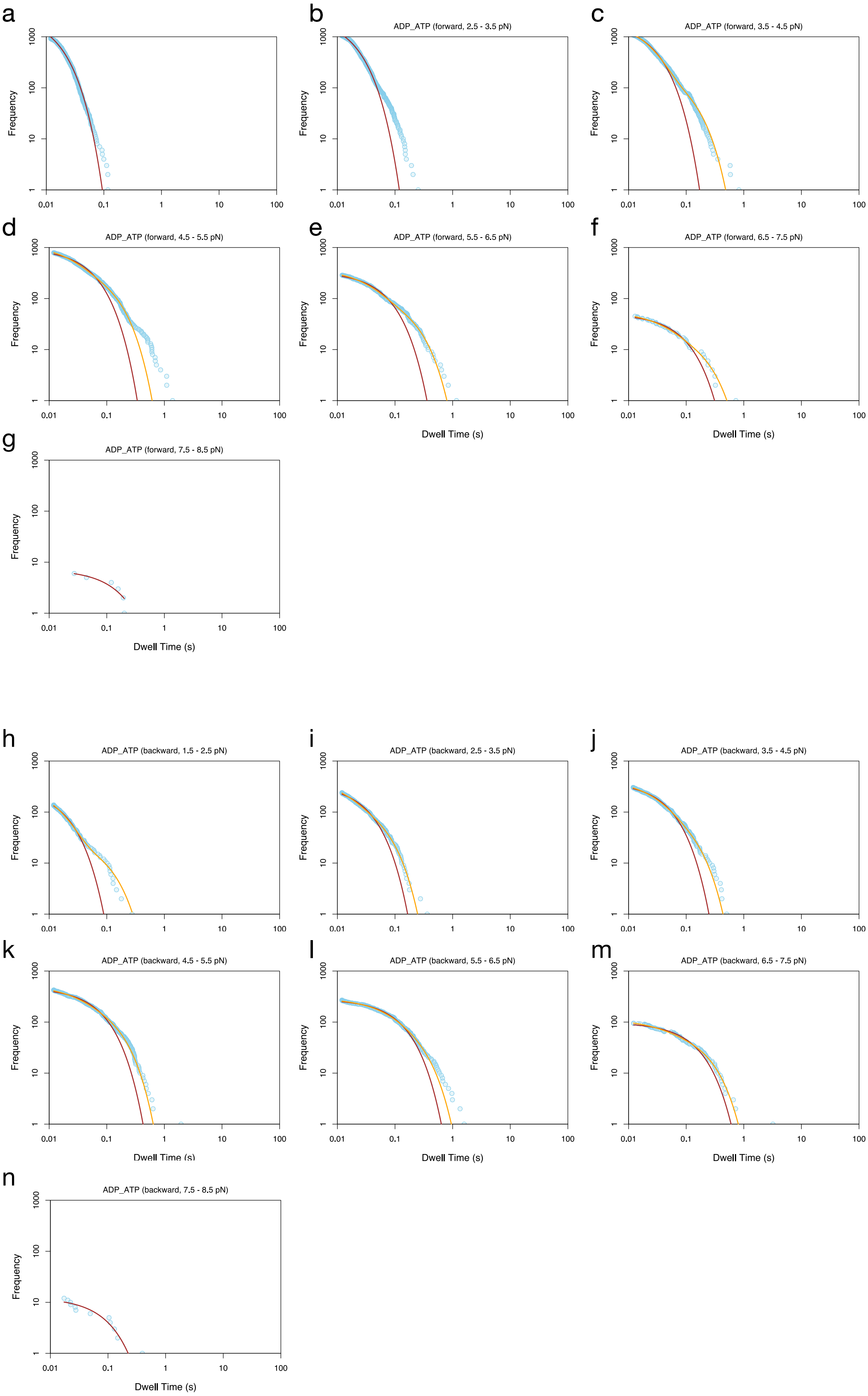
a
b
ADP_ATP (forward, 2.5 - 3.5 pN)
c
ADP_ATP (forward, 3.5 - 4.5 pN)
d
ADP_ATP (forward, 4.5 - 5.5 pN)
e
ADP_ATP (forward, 5.5 - 6.5 pN)
f
ADP_ATP (forward, 6.5 - 7.5 pN)
g
ADP_ATP (forward, 7.5 - 8.5 pN)
h
ADP_ATP (backward, 1.5 - 2.5 pN)
i
ADP_ATP (backward, 2.5 - 3.5 pN)
j
ADP_ATP (backward, 3.5 - 4.5 pN)
k
ADP_ATP (backward, 4.5 - 5.5 pN)
l
ADP_ATP (backward, 5.5 - 6.5 pN)
m
ADP_ATP (backward, 6.5 - 7.5 pN)
n
ADP_ATP (backward, 7.5 - 8.5 pN)
Frequency
1
10
100
1000
Dwell Time (s)
0.01
0.1
1
10
100

Supplementary Figure 5: Cumulative dwell-time distributions and exponential fits at 0.9 mM ATP plus 0.1 mM ADP.

Cumulative probability plots are shown for all force bins. Forward dwells (top row) and backward dwells (bottom row) were fit with single- and double-exponential models. Blue circles represent experimental data. Solid brown lines indicate single-exponential fits (y = A·exp(−b·x)), and solid orange lines indicate double-exponential fits (y = A_1·exp(−b_1·x) + A_2·exp(−b_2·x)). Fits were obtained using nonlinear least-squares regression in R. All plots are displayed on log–log axes. Parameters from both single- and double-exponential fits are: **a.** dwell = 2710.7 $e^{(-83.9*load)}$ **b.** *dwell = 2418.8* $e^{(-65.6*load)}$ **c.** dwell = 1843.5 $e^{(-44*load)}$, *dwell = 1875.7* $e^{(-59.4*load)}$ + 228.3 $e^{(-11.2*load)}$ **d.** *dwell = 946.6* $e^{(-20.3*load)}$, dwell = 746.8 $e^{(-50.1*load)}$ + *434.3* $e^{(-9.9*load)}$ ***e.*** dwell = 330.2 $e^{(-16.2*load)}$, *dwell = 266.5* $e^{(-32.8*load)}$ + 116.7 $e^{(-6.0*load)}$ **f.** *dwell = 49.5* $e^{(-12.5*load)}$, dwell = 32.9 $e^{(-28.9*load)}$ + *23.4* $e^{(-6.2*load)}$ ***g.*** dwell = 7.1 $e^{(-6.4*load)}$ **h.** dwell = 282.0 $e^{(-63.7*load)}$, dwell = 324.5 $e^{(-91.1*load)}$ + *30.2* $e^{(-12.1*load)}$ ***i.*** *dwell = 341.1* $e^{(-35.2*load)}$, dwell = 306.3 $e^{(-94.4*load)}$ + *176.5* $e^{(-21*load)}$ **j.** dwell = 383.0 $e^{(-24*load)}$, dwell = 297.9 $e^{(-41.7*load)}$ + *133.3* $e^{(-11.2*load)}$ ***k.*** *dwell = 465.3* $e^{(-14.4*load)}$, dwell = 261.5 $e^{(-39.3*load)}$ + *274.5* $e^{(-8.8*load)}$ **l.** dwell = 278.2 $e^{(-8.8*load)}$, dwell = 153.0 $e^{(-17*load)}$ + *140.9* $e^{(-5.2*load)}$ **m.** *dwell = 97.0* $e^{(-7.6*load)}$, dwell = 43.6 $e^{(-31*load)}$ + *70.0* $e^{(-5.3*load)}$ ***n.*** dwell = 12.3 $e^{(-11.2*load)}$. n for each force bin: a (1.5–2.5 pN), n = 922; b (2.5–3.5 pN), n = 1060; c (3.5–4.5 pN), n = 1091; d (4.5–5.5 pN), n = 790; e (5.5–6.5 pN), n = 287; f (6.5–7.5 pN), n = 45; g (7.5–8.5 pN), n = 6; h (1.5–2.5 pN), n = 138; i (2.5–3.5 pN), n = 240; j (3.5–4.5 pN), n = 304; k (4.5–5.5 pN), n = 426; l (5.5–6.5 pN), n = 270; m (6.5–7.5 pN), n = 95; n (7.5–8.5 pN), n = 12.

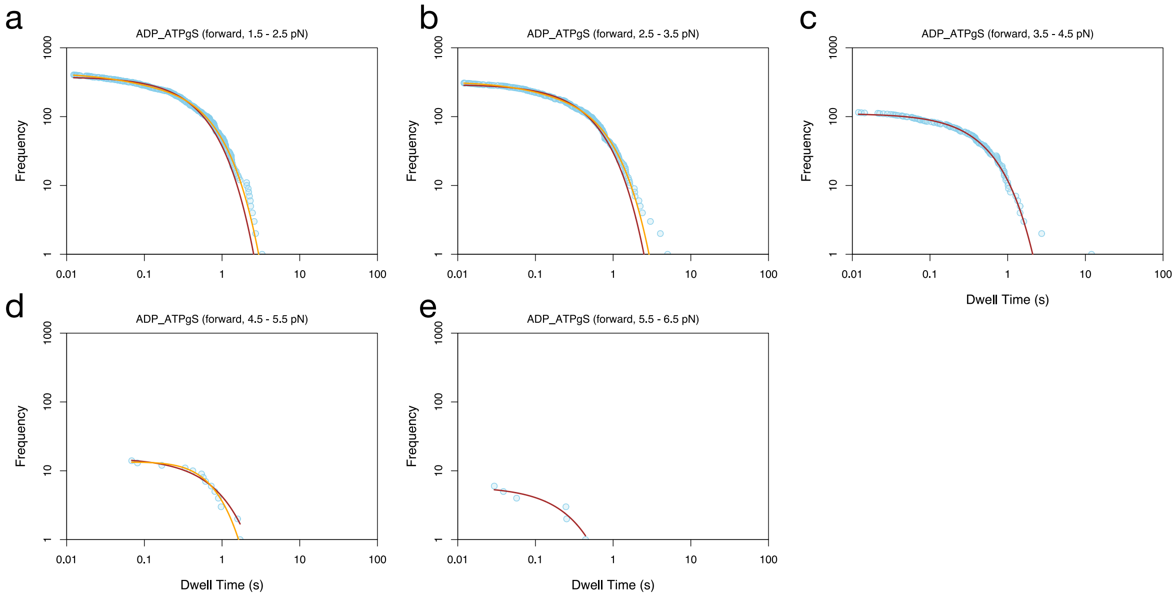
a
ADP_ATPgS (forward, 1.5 - 2.5 pN)
b
ADP_ATPgS (forward, 2.5 - 3.5 pN)
c
ADP_ATPgS (forward, 3.5 - 4.5 pN)
d
ADP_ATPgS (forward, 4.5 - 5.5 pN)
e
ADP_ATPgS (forward, 5.5 - 6.5 pN)
Frequency
1
10
100
1000
0.01
0.1
1
10
100
Dwell Time (s)

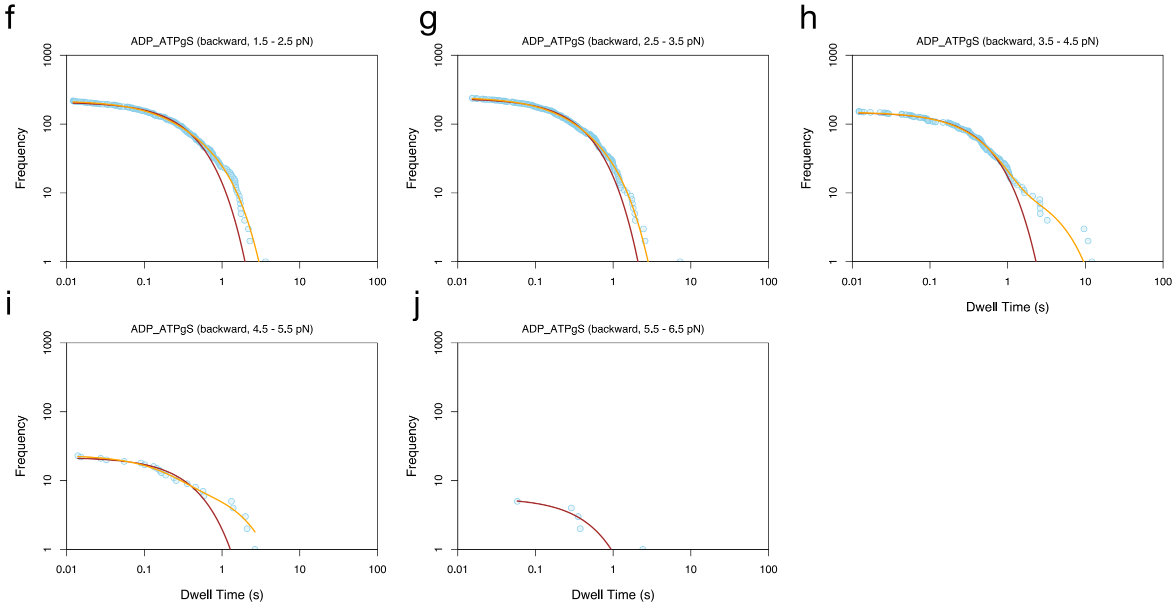
f
ADP_ATPgS (backward, 1.5 - 2.5 pN)
g
ADP_ATPgS (backward, 2.5 - 3.5 pN)
h
ADP_ATPgS (backward, 3.5 - 4.5 pN)
i
ADP_ATPgS (backward, 4.5 - 5.5 pN)
j
ADP_ATPgS (backward, 5.5 - 6.5 pN)
Frequency
1
10
100
1000
0.01
0.1
1
10
100
Dwell Time (s)

Supplementary Figure 6: Cumulative dwell-time distributions and exponential fits at 0.9 mM ATPγS plus 0.1 mM ADP.

Cumulative probability plots are shown for all force bins. Forward dwells (top row) and backward dwells (bottom row) were fit with single- and double-exponential models. Blue circles represent experimental data. Solid brown lines indicate single-exponential fits (y = A·exp(−b·x)), and solid orange lines indicate double-exponential fits ($y = A_1 \cdot \exp(-b_1 \cdot x) + A_2 \cdot \exp(-b_2 \cdot x)$). Fits were obtained using nonlinear least-squares regression in R. All plots are displayed on log–log axes. Parameters from both single- and double-exponential fits are: **a.** dwell = 378.6 $e^{(-2.3*load)}$, dwell = 97.7 $e^{(-23.1*load)}$ + 334.6 $e^{(-2.0*load)}$ **b.** dwell = 293.8 $e^{(-2.3*load)}$, dwell = 68.3 $e^{(-14.7*load)}$ + 254.8 $e^{(-1.9*load)}$ **c.** dwell = 111.2 $e^{(-2.2*load)}$, dwell = 20.7 $e^{(-29.9*load)}$ + 104.7 $e^{(-2.1*load)}$ **d.** dwell = 15.5 $e^{(-1.3*load)}$ ***e.*** dwell = 5.9 $e^{(-3.7*load)}$ **f.** dwell = 208.6 $e^{(-2.7*load)}$, dwell = 97.7 $e^{(-6.4*load)}$ + 121.9 $e^{(-1.6*load)}$ ***g.*** dwell = 236.2 $e^{(-2.6*load)}$, dwell = 98.8 $e^{(-5.9*load)}$ + 148.8 $e^{(-1.8*load)}$ **h.** dwell = 148.7 $e^{(-2.2*load)}$, dwell = 137.7 $e^{(-2.5*load)}$ + 13.2 $e^{(-0.3*load)}$ ***i.*** dwell = 21.8 $e^{(-2.4*load)}$, dwell = 14.9 $e^{(-5.8*load)}$ + 8.7 $e^{(-0.6*load)}$ **j.** dwell = 5.6 $e^{(-1.8*load)}$. n for each force bin: a (1.5–2.5 pN), n = 404; b (2.5–3.5 pN), n = 308; c (3.5–4.5 pN), n = 115; d (4.5–5.5 pN), n = 14; e (5.5–6.5 pN), n = 6; f (1.5–2.5 pN), n = 218; g (2.5–3.5 pN), n = 238; h (3.5–4.5 pN), n = 153; i (4.5–5.5 pN), n = 23; j (5.5–6.5 pN), n = 5.

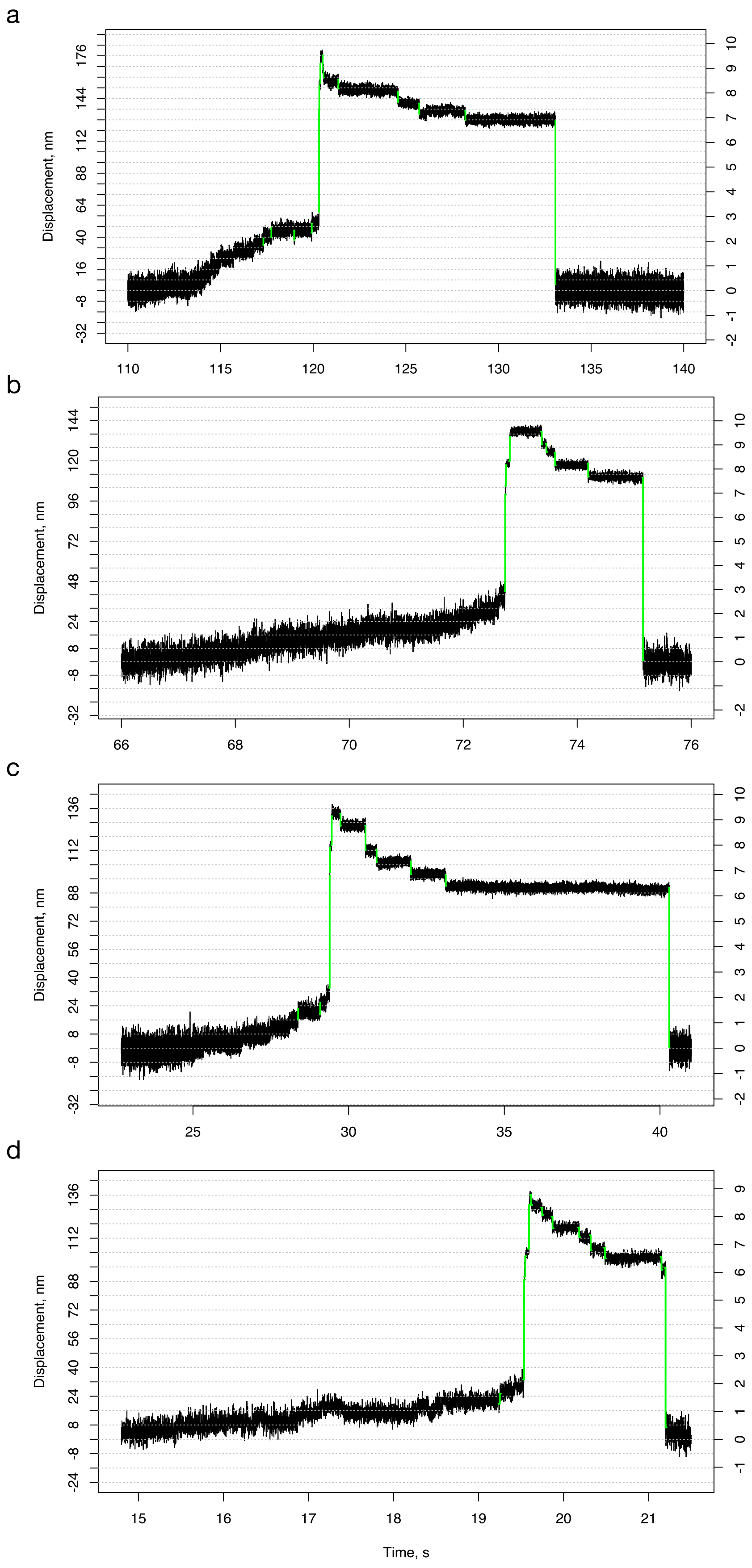
a
Displacement, nm
-32 -8 16 40 64 88 112 144 176
-2 -1 0 1 2 3 4 5 6 7 8 9 10
110 115 120 125 130 135 140
b
Displacement, nm
-32 -8 8 24 48 72 96 120 144
-2 0 1 2 3 4 5 6 7 8 9 10
66 68 70 72 74 76
c
Displacement, nm
-32 -8 8 24 40 56 72 88 112 136
-2 -1 0 1 2 3 4 5 6 7 8 9 10
25 30 35 40
d
Displacement, nm
-24 -8 8 24 40 56 72 88 112 136
-1 0 1 2 3 4 5 6 7 8 9
15 16 17 18 19 20 21
Time, s

Supplementary Figure 7: Example trapping records under superstall (SS) force at 1 mM ATPγS.

Horizontal grid lines are 8 nm apart. SS force was applied by stepping the stage at a trigger load (see Methods). Steps fitted by the t-test algorithm are shown in green. The traces show processive backsteps that are predominantly 8 nm.

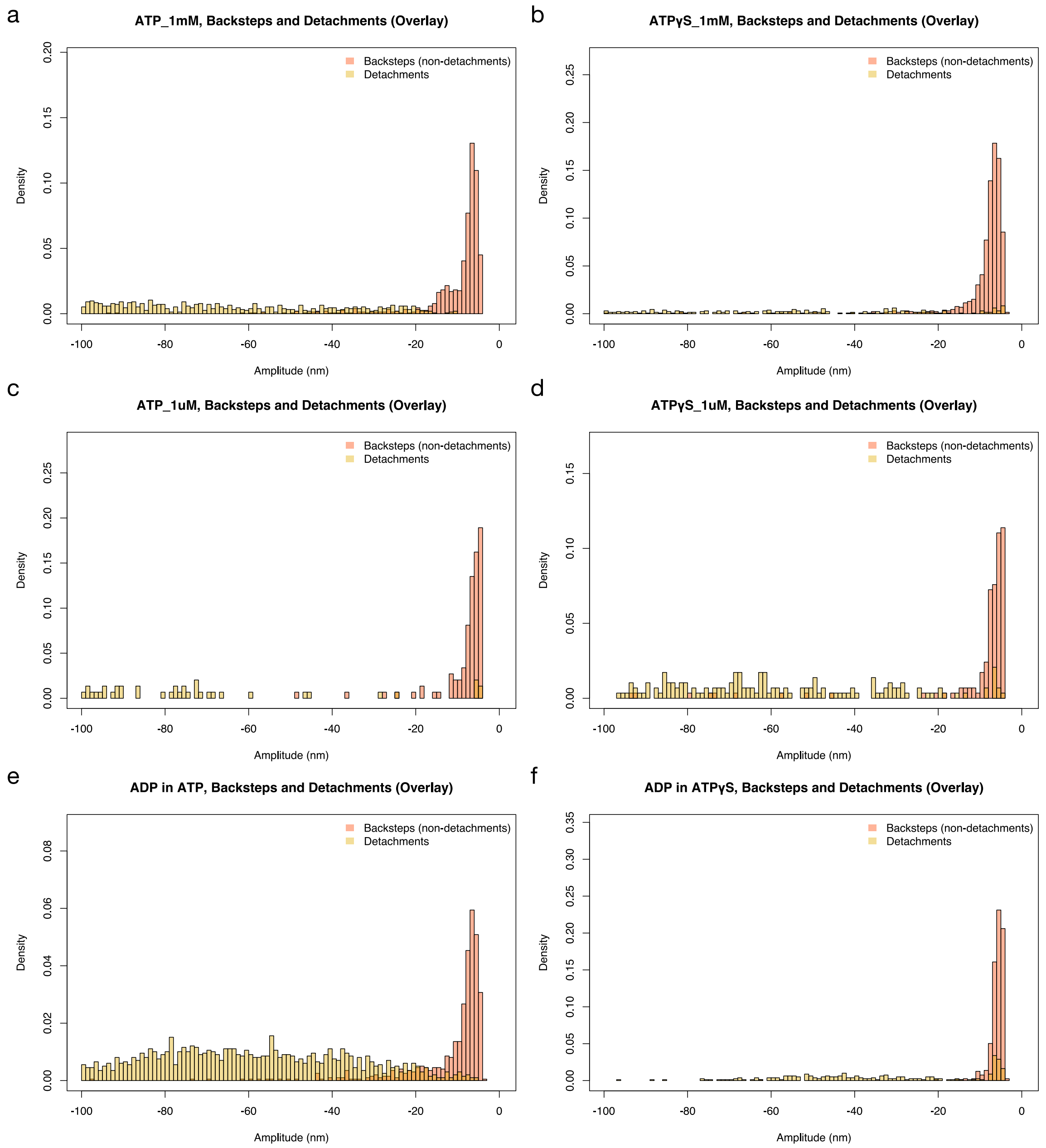

Supplementary Figure 8: Distribution of negative amplitudes partitioned into backsteps and detachments.

Overlayed probability density histograms (1 nm bins) show backsteps (orange-red) and detachments (mustard yellow) under six different conditions: (**a**) 1 mM ATP, (**b**) 1 mM ATPγS, (**c**) 1 μM ATP, (**d**) 1 μM ATPγS, (**e**) 0.9 mM ATP + 0.1 mM ADP, and (**f**) 0.9 mM ATPγS + 0.1 mM ADP. n for or each condition: a (1 mM ATP), backsteps n = 889, detachments n = 1242; b (1 mM ATPγS), backsteps n = 1101, detachments n = 463; c (1 μM ATP), backsteps n = 108, detachments n = 64; d (1 μM ATPγS), backsteps n = 142, detachments n = 156; e (0.9 mM ATP + 0.1 mM ADP), backsteps n = 657, detachments n = 1409; f (0.9 mM ATPγS + 0.1 mM ADP), backsteps n = 553, detachments n = 243.

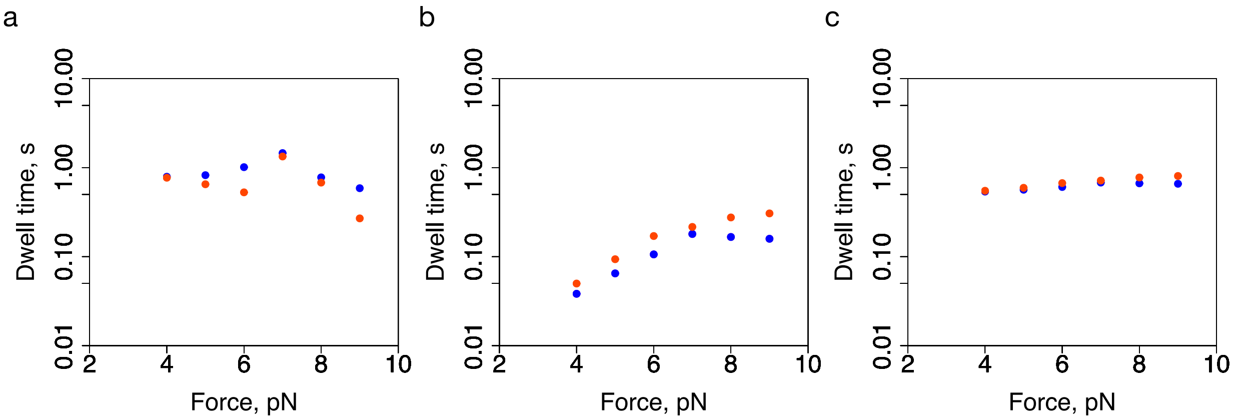

Supplementary Figure 9: Load-dependence of step dwell times in ATP versus ATPγS.

(a) Load dependence of dwell times for forward (navy blue) and backward (yellow) steps at 1 mM ATPγS. Filled circles indicate mean dwell times calculated in 1 pN bins. (b) The ATPγS data can be reproduced by adding 0.5 to the corresponding ATP dwell times (forward steps, sky blue; backward steps, red), which are overlaid for comparison.
