# Supplementary material for: ATPγS substantially defeats the biasing mechanism for kinesin steps
Source: Nat Commun. 2026 Feb 18;17:2891. doi: 10.1038/s41467-026-69573-z (PMC13031914; doi:10.1038/s41467-026-69573-z)
Supplement: Supplementary file 2 — Description of Additional Supplementary Information [file 41467_2026_69573_MOESM2_ESM.pdf]

Description of Additional Supplementary Files

File Name: Supplementary Movie 1
Description: Single backstep in 1 µM ATPγS & recover.

File Name: Supplementary Movie 2
Description: Two backsteps in 1 mM ATPγS & recover.

File Name: Supplementary Movie 3
Description: Single backslip in ATPγS induced by ADP rebinding.
