# Supplementary material for: ATPγS substantially defeats the biasing mechanism for kinesin steps
Source: Nat Commun. 2026 Feb 18;17:2891. doi: 10.1038/s41467-026-69573-z (PMC13031914; doi:10.1038/s41467-026-69573-z)
Supplement: Supplementary file 8 — Source Data [file 41467_2026_69573_MOESM8_ESM.zip › Data Availability 30Jan2026/Readme.docx]

**Figure 1.xlsx**
This file contains kinesin motility data obtained from microtubule gliding velocity measurements (nm/s), used to generate Figure 1 of the manuscript.

**Sheet 1:** Velocity at different concentrations of ATP.

**Sheet 2:** Velocity at different concentrations of ATPγS.

**Sheet 3:** Velocity at varying ratios of ATP to ATPγS.

**Figure2_and_Figure3.xlsx**
This file contains optical trap stepping data used to generate Figures 2 and 3 of the manuscript. For each experimental condition, a separate sheet is provided. Each sheet includes three columns:

1. **Experimental conditions (separate sheets):**
2. 1 mM ATP
3. 1 mM ATPγS
4. 1 µM ATP
5. 1 µM ATPγS
6. **Contents of each sheet:**
7. **Force (pN)**
8. **Amplitude (nm)**
9. **Dwell (s)**

These data were subsequently binned by force to calculate step probabilities and forward/backward (F/B) ratios (Figure 2) and load-dependent dwell times (Figure 3).

**Figure 4.xlsx**
This file contains data used to generate Figure 4 of the manuscript.

1. **Experimental conditions (separate sheets):**
2. 1 mM ATP
3. 1 mM ATPγS
4. 1 µM ATP
5. 1 µM ATPγS
6. **Contents of each sheet:**
7. **τ (s):** For each force bin (pN), the fitted mean dwell times (τ, in seconds) are provided separately for forward and backward steps. These values were obtained by fitting a single exponential decay constant to the binned dwell-time distributions. The corresponding raw distributions, fitted curves, and fit parameters are shown in Supplementary Figures S1–S6.
8. **Step amplitudes (nm):** Additional sheets report step amplitudes recorded after the stage trigger for ATP and ATPγS. Separate sheets are included specifically for the first amplitude following the trigger.

**Figure 5.xlsx**
This file contains stepping data used to generate Figure 5 of the manuscript.

1. **Experimental conditions (separate sheets):**
2. 0.9 mM ATP + 0.1 mM ADP
3. 0.9 mM ATPγS + 0.1 mM ADP
4. **Contents of each sheet:**
5. For each experimental condition, a separate sheet is provided with three columns: **Force (pN), Amplitude (nm), and Dwell (s)**. These data were used to calculate step probabilities, F/B ratios, and dwell time distributions.
6. **τ (s):** Additional sheets report the fitted mean dwell times (τ, in seconds) for each force bin, as used in panels 5e and 5k.
7. **Pre-detachment amplitudes (nm):** Separate sheets provide the amplitudes of steps immediately preceding detachment events, for both ATP and ATPγS conditions.

**Supplementary Figure 1.xlsx – Supplementary Figure 6.xlsx**
Each file contains the dwell-time data and exponential fits used to generate the corresponding supplementary figure (**Supplementary Figure** 1**- Supplementary Figure** 6).

1. **Experimental conditions:**
2. **Supplementary Figure 1.xlsx:** 1 mM ATP
3. **Supplementary Figure 2.xlsx:** 1 mM ATPγS
4. **Supplementary Figure 3.xlsx:** 1 µM ATP
5. **Supplementary Figure 4.xlsx:** 1 µM ATPγS
6. **Supplementary Figure 5.xlsx:** 0.9 mM ATP + 0.1 mM ADP
7. **Supplementary Figure 6.xlsx:** 0.9 mM ATPγS + 0.1 mM ADP
8. **Contents of each file:**
   1. **Sheets:** Each sheet corresponds to a single force bin.
   2. **Columns:** Four columns are provided:
      - **Dwell data (s):** Individual dwell times observed in that force bin.
      - **y column:** Descending rank-ordered dwell-time values used for cumulative probability analysis.
      - **Single exponential fit:** Values from fitting the data to a single exponential decay model.
      - **Double exponential fit:** Values from fitting the data to a double exponential decay model.

These files provide the raw and fitted data underlying the cumulative probability plots and exponential fits shown in Supplementary Figures 1-6.

**Supplementary Figure 8.xlsx**
This file contains the backstep and detachment amplitude data used to generate Supplementary Figure 8.

1. **Experimental conditions (separate sheets):**
   1. 1 mM ATP
   2. 1 mM ATPγS
   3. 1 µM ATP
   4. 1 µM ATPγS
   5. 0.9 mM ATP + 0.1 mM ADP
   6. 0.9 mM ATPγS + 0.1 mM ADP
2. **Contents of each sheet:**
   - **Backstep (nm):** Amplitudes of observed backsteps in that condition.
   - **Detachment (nm):** Amplitudes of observed detachments in that condition.

These datasets were used to generate the overlaid probability density histograms of negative amplitudes shown in Supplementary Figure 8.

**Supplementary Figure 9.xlsx**
This file contains the averaged dwell-time data used to generate Supplementary Figure 9.

1. **Experimental conditions:**
2. 1 mM ATP
3. 1 mM ATPγS
4. **Contents of each sheet:**
   - **Force bin (pN)**
   - **Average dwells (s), forward:** Mean forward step dwell times in that force bin.
   - **Average dwells (s), backward:** Mean backward step dwell times in that force bin.

These averaged dwell times were calculated from the data provided in Figure2_and_Figure3.xlsx. The comparison shown in Supplementary Figure 9c was generated by adding **+0.5 s** to the ATP dwell times; this offset is not included as a separate sheet but can be reproduced directly from the ATP data provided here.

**Note on raw optical trap traces**
For Figures **1, 5, and Supplementary Figure 7**, the underlying raw optical trap trace files are too large to include here. These datasets are available from the corresponding author upon reasonable request.
